# Commenting on biomarkers for differentiating between SIRS and sepsis

**DOI:** 10.4103/0974-2700.66554

**Published:** 2010

**Authors:** Viroj Wiwanitkit

**Affiliations:** Wiwanitkit House, Bangkhae, Bangkok Thailand - 101 60

Sir,

I read the recent report on new biormarkers for differentiating between systemic inflammatory response syndrome (SIRS) and sepsis with great interest.[1] Indeed, at present, there are many new biomarkers that might be useful in the clinical management of cases suspected for sepsis.[2] Punyadeera *et al*. reported that metalloproteinases and selectin play a role in the distinction between SIRS and sepsis, and that IL-1α, IP-10, sTNF-R2 and sFas appear to be indicative for the progression from sepsis to septic shock.[1] Based on laboratory medicine principle, there are some comments on this work. First, as Punyadeera *et al*. mentioned that their results were only preliminary data, the main flaw of this work is the very few numbers of subjects studied. Second, there can be several factors that affect the level of studied parameters. Several parameters are classified as respondents to inflammation and there is no data whether there is any concomitant inflammation in the studied subjects. In addition, in sepsis, the causative agents are still questionable. Whether different causative agents for sepsis result in different levels of studied parameters needs to be clarified. Finally, as noted by Herzum and Renz, international definition and staging criteria have to be internationally agreed and set to be used in the interpretation of the laboratory parameters in real clinical practice.[3]
